# Nurses' Level of Knowledge on Management of Preeclampsia / Eclampsia and the associated factors in Northern Tanzania: An Analytical Cross-Sectional Study

**DOI:** 10.24248/eahrj.v7i1.708

**Published:** 2023-07-12

**Authors:** Wemael Wilfred Mkumbo, Fabiola V. Moshi

**Affiliations:** a Department of Clinical Nursing, School of Nursing and Public Health, the University of Dodoma, Dodoma, Tanzania; b Department of Nursing Management and Education, School of Nursing and Public Health, the University of Dodoma, Dodoma, Tanzania.

## Abstract

**Background:**

Preeclampsia/Eclampsia is among the hypertensive disorder of pregnancy. It is accompanied by high blood pressure, protein in the urine, convulsion, and sometimes oliguria. This condition results in higher maternal morbidity and mortality worldwide.

**Objectives:**

The objective of this study was to assess nurses' level of knowledge and factors influencing nurses' knowledge of managing preeclampsia/Eclampsia in Northern Tanzania.

**Method:**

The study was analytical cross-sectional study design. A total sample of 176 nurses working in the maternity block was enrolled in the study. A census sampling technique was used to get 176 nurses. A closed-ended structured questionnaire was used to collect data. Statistical Package for the Social Sciences (SPSS) version 26 was used for data analysis. Knowledge was categoried into low and high knowledge, two, less than 50% had low knowledge and above 50% had high knowledge. Inferential analysis using a logistic regression model was used to establish factors associated with knowledge.

**Results:**

The study revealed that more than half of interviewed nurses 129(73.3%) had high knowledge while 47(26.7%) had low knowledge on management of preeclampsia/Eclampsia. After controlling for confounders, factors associated with knowledge were nurse who got On job training on Preeclampsia/Eclampsia management.

**Conclusions:**

Some essential predictors of knowledge were shown among nurses, but generally, knowledge about the management of preeclampsia/eclampsia among nurses was high. Managing women with preeclampsia/eclampsia and their fetuses, there is a great need for advanced strategies to increase knowledge about the management to nurses.

## BACKGROUND

Maternal death is among the significant public health problems, whereby hypertensive disorders of pregnancy, including Preeclampsia/Eclampsia, raise the number of maternal and fetal morbidity and mortality worldwide. These disorders are reported to be the second leading cause of maternal mortality.^1^ Preeclampsia is a cardiovascular condition causing endothelial malfunction and vasospasm after 20 weeks gestation and can occur as late as 4-6 weeks after delivery. Preeclampsia is the occurrence of high blood pressure in pregnancy after 20 weeks, whereby diastolic blood pressure rises ≥ 90 mm Hg and Considerable protein in urine > 0.3g/24 hours^2^ Preeclampsia with severe features may present with a blood pressure of 160/110 mm Hg or more. low platelet count, renal failure, sudden onset of headache which does not respond to medication, and visual impairment.^3^

Additionally, it accounts 14% of maternal death globally, 22,000 maternal death in Asia, 3800 maternal death in Latin America and 150 in the Caribbean, while sub-Saharan Africa account about 16.0%, besides yearly report 25,000 maternal death in Africa, data from world health organization.^4^ In Ethiopia, the prevalence of pregnancy-induced hypertension is 2.2% to 18.3% whereby accounts for 19% of deaths.^4^

In Tanzania, the maternal mortality ratio is high as 556 deaths per 100,000 live births^5^ whereby deaths due to eclampsia are 35%, in northern Tanzania accounts for 28 %.^6^ In Kilimanjaro cause, 18% of Maternal deaths.^7^

According to Tanzania, maternal and perinatal surveillance sets design that when maternal demise occurs should be reported within 48 hours and assessment within seven days to recognize the cause of death, regardless of all design the number of maternal demises still increasing by eight times in 2018 from 2017 in Dodoma region.^8^ Thus increasing the number of pregnancy-induced hypertension leads to more referral cases.^8^ The study reveals that the maternal mortality rate remains high in developing countries, primarily due to deprived access to prenatal care, delay in managing a woman with hypertensive disorder of pregnancy, and inadequate service provider skills.^9^

Preeclampsia/eclampsia can lead to poor growth of the baby during intrauterine life. This accounts for 30%, inadequate weight and delivered before time, were by 73.3%.^10^ The reason for the occurrence of complications revealed by other studies was a lack of knowledge and skills in diagnostic measures like urinalysis for diagnostic purposes. Also, they identified that complication occurs as a result of inadequate knowledge, undesirable attitude toward hypertension in pregnancy, poor awareness, decision, and treatment.^4^

Moreover, the management of the woman with preeclampsia/eclampsia depends on the following factors such as the severity of the disease, gestational age, maternal and fetal condition.^11^ The nurse to identify all of these must possess the required knowledge to overcome the situation.

In Tanzania, the MoHCDGEC works in collaboration with health partners (to provide training and workshops on BEmONC and CEmONC, but still, pre/eclampsia is the second after PPH cause of maternal deaths in the country.

The study revealed that nurses have insufficient knowledge of the management of pre-eclampsia/eclampsia as evidenced by a study done in Egypt which reported that 30% of nurses had poor knowledge of how to care for the pregnant woman with fit following eclampsia.^1^ Also, a study was done in rural Karnataka State, India, revealed insufficient common knowledge.^12^ There was no data on the level of nurses' knowledge on the management of Preeclampsia/Eclampsia in Northern Tanzania. The study aimed to assess the level of knowledge to nurses working in an obstetric unit in the Kilimanjaro region

## METHODS

### Study design and Target Population

This study presents an analysis of nurses' knowledge on the management of Preeclampsia/Eclampsia. The study was aimed to assess nurses' level of knowledge and factors influencing nurses' knowledge on the management of preeclampsia/Eclampsia. Study respondents were nurses working in the obstetric unit

### Study Setting

The study was conducted in northern Tanzania. The selection of the region was based on the fact that preeclampsia/Eclampsia is the among direct cause of maternal death in the region but also no study ever conducted in the region concerning Preeclampsia/Eclampsia management to nurses on facility basis,as nurses are the one who stay with the patient for longer time compared to other medical personel. The Kilimanjaro region is located in the Northern zone of Tanzania, which consists of 6 districts named Rombo, Mwanga, Same, Siha, Hai, and Moshi, whereby Moshi is divided into Moshi Municipal and Moshi district. The region has 13,209 km^2^ and 1,376,702 population. Kenya borders the region to the north and east. The Kilimanjaro region borders Tanga Region to the south. The southwest by the Manyara Region, and the west by the Arusha Region.

This study employed a cross-sectional study. Under cross-sectional design, the variables in a sample were studied only once to conclude their relationships. The study applied a survey in a cross-sectional design, which uses questionnaires to seek information from respondents at one point in time.^13^ The choice of cross-section design was based on the fact that the design helped to provide the information concerning the relationship between participant variables and factors influencing nurses' knowledge in the management of preeclampsia/eclampsia. Furthermore, the design fits the research question that was studied hence enable the researcher to get a clear picture of the existing situation and assess the relationship between variables and the factors influencing nurses' knowledge in the management of preeclampsia/eclampsia in a single study.

### Sample Size Calculation and Sampling Technique

### Sample Size Calculation

Cochran formula (1977) was used to compute the sample size for the study.



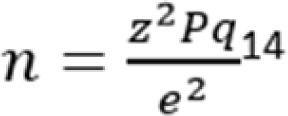



Where:

n = minimal sample size

e = the desired level of precision [marginal error] 5%

P=11.8% which is proportional to Knowledge on Prevention and Management of Preeclampsia and Eclampsia among Nurses in the health facility which was obtained from the study done in Dodoma region Tanzania^1^ with a formula obtained from Charan, J and Biswas.^14^

q = 1-P

Z = confidence interval 1.96 = 95%



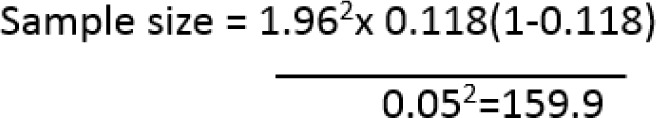



The minimum required sample size was 160, and I added 10% for non-response.

The total sample size was 176 nurses

### Sampling Technique

The study involved seven councils of the Kilimanjaro region, whereby all seven council hospitals, regional hospital, and consultant(zonal) hospital of the region were selected conveniently (a referral point for eclampsia cases), the random sampling technique was used to select health center whereby one hearth center was selected randomly in each council, the following health center was selected Keni, Charlotte, Hedaru, Kisangara, Himo OPD, Kisiki and Majengo Furthermore, census sampling were used to get nurses who are working in an obstetric unit in all health facilities selected.

### Data Collection Tool

A structured questionnaire was used for data collection. The instruments used for this study were adapted from Olaoye^15^ and modified. The modification was done to the question observed not clear or does not suit the need for the current study. The pre test was done to the instrument to a total of 17 participants at Makole health centre for clarity of the tool, after pre test the instrument was revised and finalized for use in data collection. The instrument consists of two parts; part 1: Background information, part 2: Knowledge of management of preeclampsia/eclampsia. Whereby 40 questions were used to measure knowledge. The major themes used in the knowledge questions were nurses' knowledge on the management of preeclampsia/Eclampsia, drugs used to manage patients with preeclampsia/eclampsia, and dosage of magnesium.

### Data Collection Procedure

The data were collected by using self-administered questionnaire. Datum was collected by two trained assistant researchers and a principal researcher. The participant answered questionnaires; the filled questionnaires were collected by the researcher or a trained researcher assistant. The duration for data collection was four weeks.

### Variable and how the Variable was Measured

### Knowledge

The study measured knowledge by asking 40 close-ended questions(multiple choice); every question comprised 1 point, so the total score was 40. Moreover, The score was categorized into two those scored less than 50% were termed as having low knowledge and those with more than 50% having high knowledge

### Data Analysis

The collected data were coded and entered into SPSS version 26 to obtain the results of the study. The data analysis involved descriptive statistics in computing means, standard deviations, frequencies, percentages, and linear regression. There is simple and multiple logistic regression to find predictors of knowledge. In simple logistic regression, the aim was to find the specific predictors of knowledge. After was computed whereby all variables with a *p-Value* of < .05 had a significant relationship with nurses' knowledge. Moreover, multiple logistic regression was computed to variables with a p-Value of less than 0.2 for the adjustment of confounders; again, the variable with a p-value of <0.05 decided to be having a significant relationship with nurses' knowledge.^13^

### Ethical Consideration

The ethical clearance for this study was obtained from the Health Research Ethics Committee of the University of Dodoma. The researcher also obtained a permission letter from the authority at the University of Dodoma and RAS Kilimanjaro Region, the permission made by the university on behalf of the government with code number MPEC/R/10/1 dated 4^th^ july 1980 which empower the university vice chancellor to issue research clearance, the clearance letter were obtained on 23^rd^ November 2020 where the study was conducted. The ethical clearance letter attached as picture. An individual informed consent was attained from study applicants after full enlightenment of the study goals and processes. Privacy and confidentiality were guaranteed to all study respondents.

## RESULTS

### Socio-demographic Characteristics of Study Respondent

Sociodemographic characteristics of study participants: A total of 176 nurses were included in this study, with a response rate of 100%. The mean age of study participants was 35.70 years with a standard deviation of 10.64 years. The minimum age is = 21, and the maximum age is = 67. The majority of the participants' 83(47.2%) age was range from 26-35 years. Concerning the professional training majority of study participants, 96 (54.5%), were nurses with the diploma. Concerning years in the nursing profession, 55 (31.3%) was having experience of more than 12 years. Concerning the level of the facility, more than half of the respondents, 91(51.7%), were from district hospitals. The majority of nurses, 137 (77.7%), were not attended on the job training. More information concerning the sociodemographic of study respondents is indicated in Table 1

**TABLE 1:** Sociodemographic Characteristics of Study Respondents

Variable	Frequency (n)	Percent (%)
Age category		
Less than 25 years	23	13.1
26–35	83	47.2
36 and above	70	39.8
Gender		
Male	34	19.3
Female	142	80.7
Marital status		
Widowed	4	2.3
Married	102	58
Not married	70	39.8
Level of professional training		
Certificate in Nursing	44	25
Diploma in nursing	96	54.5
BSc Nursing and above	36	20.5
Years in the nursing profession		
Below three years	54	30.7
4 to 7 years	44	25
8 to 11 years	23	13.1
Above 12 years	55	31.3
Level of facility		
District hospital	91	51.7
Health center	49	27.8
Consultant hospital	18	10.2
Regional Referral Hospital	18	10.2
Type of health facility		
Government	112	63.6
Non-Government	64	36.4
Years working (experience) in the obstetric unit		
Below three years	48	27.3
4 to 7 years	77	43.8
8 to 11 years	22	12.5
Above 12 years	29	16.5
Orientation newly assigned in Obstetric		
Yes	114	64.8
No	62	35.2
Number of nurses in Obstetric units		
< 10	49	27.8
10 to 20	91	51.7
31 to 40	18	10.2
above 41	18	10.2
On the job training (workshop)		
Yes	39	22.2
No	137	77.8
Observing colleagues		
Yes	25	14.2
No	151	85.8
Job Aid reference		
Yes	38	21.6
No	138	78.4

### Nurses' Knowledge Description on Management of Eclampsia/Preeclampsia

The maximum score was 39 points, while the lowest was 11 points with a variance of 30.75. The respondents' mean score for knowledge of preeclampsia/Eclampsia was 23.52 ±5.54. More than a half 91(51.7%) of respondent they knew the meaning of eclampsia. The majority of respondents, 158(89.8%), know the drug of choice to control the eclamptic fit. The majority of respondents, 139(79%), know a drug to give a woman with eclampsia when diastolic remains 110mmmHg after management. Most of the respondents 161(91.5%), know that an Early ANC visit is vital in preventing preeclampsia/Eclampsia. The majority of respondents, 161(91.5%), know that if the woman gets eclampsia, she/he should shout for help. For more information concerning the description of the respondent, knowledge see Table 2

**TABLE 2:** Description of Respondent Knowledge Items

knowledge of preeclampsia/Eclampsia	Incorrect n (%)	Correct n (%)
What is eclampsia	85 (48.3)	91 (51.7)
What is the drug of choice for the management of eclamptic fit?	18 (10.2)	158 (89.8)
What is the dosage of magnesium sulfate loading dose?	111 (63.1)	65 (36.9)
What is the dosage of magnesium sulfate for maintenance dose?	83 (47.2)	93 (52.8)
What drug patient should be given if diastolic pressure remains 110mmHg or more?	37 (21)	139 (79)
What causes the fetus to suffer from Pre-eclampsia/eclampsia?	75 (42.6)	101 (57.4)
What is the sign of eclampsia?		
Visual disturbance	58 (33)	118 (67)
Convulsion	56 (31.8)	120 (68.2)
Low urine output or production	105 (59.7)	71 (40.3)
Does chronic hypertension predispose to eclampsia?	36 (20.5)	140 (79.5)
Does high body weight compare to height predispose to eclampsia?	111 (63.1)	65 (36.9)
Does carrying more than one fetus in a womb predispose to eclampsia?	124 (70.5)	52 (29.5)
Does a woman above 40 years of age predispose to eclampsia	86 (48.9)	90 (51.1)
Does Eclampsia lead to Cardiac failure?	87 (49.4)	89 (50.6)
Does Eclampsia lead to Visual loss?	114 (64.8)	62 (35.2)
Does Eclampsia lead to Postpartum haemorrhage?	59 (33.5)	117 (66.5)
Does Eclampsia lead to Early separation of the placenta?	82 (46.6)	94 (53.4)
Does attending the antenatal clinic as indicated prevent Eclampsia?	15 (8.5)	161 (91.5)
Does the use of an aspirin supplement prevent Eclampsia?	140 (79.5)	36 (20.5)
Does reducing body weight prevent Eclampsia?	128 (72.7)	48 (27.3)
Does doing light exercises prevent Eclampsia?	125 (71)	51 (29)
Does shout for help needed during fit?	15 (8.5)	161 (91.5)
Does ensure the woman's airway is open needed during fit?	65 (36.9)	111 (63.1)
Does giving an antihypertensive drug needed during fit?	42 (23.9)	134 (76.1)
What care should be provided to a woman after convulsion?		
Should I give oxygen 4–6 litres per minute by mask if available?	105 (59.7)	71 (40.3)
Should I observe colour for cyanosis and the need for oxygen?	33 (18.8)	143 (81.3)
Should I Aspirate the mouth and throat as necessary?	77 (43.8)	99 (56.3)
What physical examination is needed to the patient after convulsions/fits	63 (35.8)	113 (64.2)
What is the recommended intravenous line for managing eclampsia?	42 (23.9)	134 (76.1)
Which sign/symptoms differentiate a patient with Eclampsia from other hypertensive disorders?	88 (50)	88(50)
What is the sign of magnesium toxicity?	42 (23.9)	134 (76.1)
Should I assess respiratory rate? Is the respiratory rate at least 16 per minute?	81 (46)	95 (54)
Should I assess patellar reflexes? Are Patellar reflexes present?	42 (23.9)	134 (76.1)
Should I Assess urinary output? Is Urinary output at least 30 mL per hour over the preceding four hours?	95 (54)	81 (46)
Does a woman with Eclampsia given magnesium sulfate after delivery?	26 (14.8)	150 (85.2)
If yes, for how long women with eclamptic fit should be given magnesium sulfate?	63 (35.8)	113 (64.2)
What is the immediate measure in case of magnesium toxicity?		
Can I Withhold or delay the drug?	111 (63.1)	65 (36.9)
Can I assess ventilation?	136 (77.3)	40 (22.7)
Can I give Calcium Gluconate 1gm (10 ml in 10% solution)	32 (18.2)	144 (81.8)
Can I give hydralazine?	8 (4.5)	168 (95.5)

**FIGURE 1:**
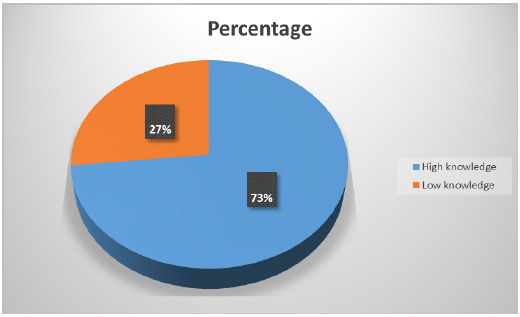
Categories of Knowledge

The knowledge mean score was 23.52, the maximum score was 39 points, while the lowest was 11 points with a variance of 30.75. When the scores were categorized, the study showed that the respondents, 129(73.3%) had high knowledge of the management of preeclampsia/eclampsia while 47(26.7%) had low knowledge of preeclampsia/Eclampsia management as shown in figure 1

### The Relationship between Nurses' Characteristics and Knowledge on Preeclampsia/Eclampsia

Variables which showed significant relationship with knowledge were level of health facility a nurse is working (p=0.015), type of health facility (*p=0.001*), number of nurses in the unit (p=0.015), number of patients served per day (*p=0.001*) and ever received on job training (*p=0.018*) Table 3

**TABLE 3:** The Relationship Between Nurses' Characteristics and Knowledge on Pre-Eclampsia/Eclampsia

Variable	Inadequate knowledge n (%)	Adequate knowledge n (%)	X^2^	P-Value
Age of respondents			1.376	0.503
Less than 25 years	5 (21.7)	18 (78.3)		
26–35	20 (24.1)	63 (75.9)		
36 and above	22 (31.4)	48 (68.6)		
Gender			0.805	0.369
Male	7 (20.6)	27 (79.4)		
Female	40 (28.2)	102 (71.8)		
Marital status			1.485	0.476
Widowed	2 (50)	2 (50)		
Married	25 (24.5)	77 (75.5)		
Not married	20 (28.6)	50 (71.4)		
Level of professional training			4.54	0.103
Certificate in Nursing	17 (38.6)	27 (61.4)		
Diploma in nursing	23 (24)_	73 (76)		
BSc Nursing and above	7 (19.4)	29 (80.6)		
Years in the nursing profession			1.774	0.621
Below 3 years	11 (20.4)	43 (79.6)		
4 to 7 years	12 (27.3)	32 (72.7)		
8 to 11 years	7 (30.4)	16 (69.6)		
Above 12 years	17 (30.9)	38 (69.1)		
Level of health facility			10.396	0.015
Health center	24 (26.4)	67 (73.6)		
District hospital	19 (38.8)	30 (61.2)		
Regional Referral Hospital	0 (0)	18 (100)		
Consultant hospital	4 (22.2)	14 (77.8)		
Type of health facility			10.367	0.001
Government	39 (34.8)	73 (65.2)		
Non-Government	8 (12.5)	56 (87.5)		
Years of work (experience) in obstetric unit			2.036	0.565
Below 3 years	13 (27.1)	35 (72.9)		
4 to 7 years	17 (22.1)	60 (77.9)		
8 to 11 years	7 (31.8)	15 (68.2)		
Above 12 years	10 (34.5)	19 (65.5)		
Newly graduate nurse or those shifted from other unit within 3 months.			3.769	0.052
Yes	25 (21.9)	89 (78.1)		
No	22 (35.5)	40 (64.5)		
Number of nurses in obstetric unit			10.396	0.015
< 10 nurses	19 (38.8)	30 (61.2)		
10 to 20 nurses	24 (26.4)	67 (73.6)		
21 to 40 nurses	4 (22.2)	14 (77.8)		
41 nurses	0 (0)	18 (100)		
Number of patients served per day			16.564	0.001
Below 10	12 (42.9)	16 (57.1)		
20–30	15 (44.1)	19 (55.9)		
40–50	18 (21.7)	65 (78.3)		
Above 60	2 (6.5)	29 (93.5)		
Having training on Preeclampsia/Eclampsia Got On Job training workshop			4.934	0.018
Yes	5 (12.8)	34 (87.2)		
No	42 (30.7)	95 (69.3)		

### Factors Associated with Nurses' Knowledge on Management of Preeclampsia / Eclampsia

Before adjustment for confounder, the following variables were statistically significant were, type of the facility [Non government (OR =3.74 at 95%CI =1.62-8.633, P <0.002)], number of patient served per day [40 to 50 patients(OR =2.708 at 95% CI =1.087-6.745, P<0.032)], again number of patient served per day [60 and above patients(OR =10.875 at 95% CI =2.159-54.766, P<0.004)] and nurse who got training/workshop of management of preeclampsia/Eclampsia [ On job training (OR =0.333 at 95% CI =0.122-0.91,*P<0.032*)]. (Table 4)

**TABLE 4:** Predictors of Nurses' Knowledge on the Management of Preeclampsia and Eclampsia Before Adjusting for Confounders

Univariate analysis				
Variables	OR	CI		p-value
		Lower	Upper	
Level of professional training				
Certificate in Nursing	1			
Diploma in nursing	1.998	0.928	4.302	0.077
BSc Nursing and above	2.608	0.936	7.266	0.067
Level of health facility				
Health center	1			
District hospital	0.566	0.27	1.185	0.415
Regional Referral Hospital	578677556	0		0.131
Consultant hospital	1.254	0.376	4.184	0.998
Type of health facility				
Government	1			
Non-Government	3.74	1.62	8.633	0.002
Newly graduate nurse or those shifted from other unit with in 3 months.				
Yes	1			
No	0.511	0.258	1.012	0.054
Number of nurses in obstetric unit				
< 10 nurses	1			
10 to 20 nurses	1.768	0.844	3.706	0.131
21 to 40 nurses	2.217	0.634	7.745	0.212
41 nurses	1.023E+09	0		0.998
Number of patients served per day				
Below 10	1			
20–30	0.95	0.346	2.606	0.921
40–50	2.708	1.087	6.745	0.032
Above 60	10.875	2.159	54.766	0.004
Having training on Preeclampsia/Eclampsia Got On Job training workshop				
Yes	1			
No	0.333	0.122	0.91	0.032

### Factors Associated with Knowledge on the Management of Preeclampsia/Eclampsia after Adjustment for Confounders

After adjustment for confounder, the variable which were statistically significant was, nurses' who got on training/workshop of management of preeclampsia / Eclampsia [ On job training (OR =0.333 at 95% CI =0.122 -0.91, P<0.032)], the following were confounders for knowledge on managing preeclampsia/eclampsia were level of professional training and newly graduate nurse or those shifted from other unit to get orientation or training on care of preeclampsia/eclampsia patient and other obstetric condition they were considered as confounders as they can contribute to the factors by 80% see Table 5 for more information

**TABLE 5:** Factors Associated with Knowledge on the Management of Preeclampsia/Eclampsia After Adjustment for Confounders

Multivariate analysis				
Variables	AOR	CI		p-value
		Lower	Upper	
Level of professional training				
Certificate in Nursing	1			
Diploma in nursing	1.415	0.603	3.322	0.425
BSc Nursing and above	2.673	0.854	8.36	0.091
Type of health facility				
Government	1			
Non-Government	2.747	0.868	8.693	0.086
Newly graduate nurse or those shifted from other unit				
Yes	1			
No	1.259	0.511	3.103	0.617
Number of patients served per day				
Below 10	1			
20–30	1.203	0.38	3.815	0.753
40–50	2.484	0.927	6.652	0.07
Above 60	5.407	0.862	33.92	0.072
Having training on Preeclampsia/Eclampsia Got On Job training workshop				
Yes	1			
No	0.317	0.106	0.946	0.039

## DISCUSSION

The study found that A total of 129(73.3%) of nurses had high knowledge on management of pre-eclampsia/ eclampsia while 47(26.7%) had low knowledge This implies that more than half of the respondents had high knowledge on management of preeclampsia/ eclampsia Similarly study done In Tanzania reported that nurses had adequate knowledge.^1^

Concomitantly Inadequate knowledge of service provider on management was also reported in a previous study in Democratic republic of Congo.^16^ Again, a similar study done in Bujumbura reviels knowledge gap pertaing to hypertension in pregnancy among health care providers^17^ moreover study conducted in Zanzibar reviels inadequate knowledge of service providers.^18^ The reason for deference in results could be; geographical location, sample size, method of analysis and type of respondents.

Moreover study also showed those who get on job training as statistically significant, this was similar to study done in Zanzibar showed those get on job training had knowledge.^18^ This was similar to a study conducted in Dar-es-Salaam–Tanzania, Bangladesh, and Nigeria, the result showed those who did not get on-job training have less knowledge than those who get training. Possibly the reason for similarities could be geographical location.^19,15,20^

## CONCLUSION

This study reveals high knowledge on the management of preeclampsia/Eclampsia among nurses working in the Kilimanjaro region, Tanzania. Moreover, it revealed that nurses who had on job training on management of preeclampsia /Eclampsia were predictor of knowledge among nurses. These findings reveal a need for Further research to be undertaken using a mixed approach to explore factors influencing nurses' knowledge and perceived barriers to managing preeclampsia/ Eclampsia in the Kilimanjaro region.

For advanced educational strategies to raise knowledge on the management of preeclampsia/eclampsia among nurses.

### Limitation of the study

The data collection was done for four weeks to obtain data. During data collection, respondents were busy with patient care, which led the researcher and assistant researcher to wait until they finish their activaties. To overcome this limitation, the researcher decides to be tolerant to adhering to the timetable of respondents. this made the researcher collect the data beyond the time given
